# Pleiotropy of the *de novo*-originated gene *MDF1*

**DOI:** 10.1038/srep07280

**Published:** 2014-12-02

**Authors:** Dan Li, Zhihui Yan, Lina Lu, Huifeng Jiang, Wen Wang

**Affiliations:** 1 State Key Laboratory of Genetic Resources and Evolution, Kunming Institute of Zoology, Chinese Academy of Sciences (CAS), Kunming, Yunnan 650223, People's Republic of China; 2 Key Laboratory of Systems Microbial Biotechnology, Tianjin Institute of Industrial Biotechnology, Chinese Academy of Sciences(CAS), Tianjin 300308, People's Republic of China

## Abstract

*MDF1* is a young *de novo*-originated gene from a non-coding sequence in baker's yeast, *S. cerevisiae*, which can suppress mating and promote vegetative growth. Our previous experiments successfully demonstrated how Mdf1p binds to the key mating pathway determinant MATα2 to suppress mating. However, how Mdf1p promotes growth and fulfills the crosstalk between the yeast mating and growth pathways are still open questions. Thus, the adaptive significance of this new *de novo* gene remains speculative. Here, we show that Mdf1p shortens the lag phase of *S. cerevisiae* by physically interacting with *SNF1*, the governing factor for nonfermentable carbon source utilization, and thereby confers a selective advantage on yeasts through the rapid consumption of glucose in the early generational stage in rich medium. Therefore, *MDF1* functions in two important molecular pathways, mating and fermentation, and mediates the crosstalk between reproduction and vegetative growth. Together, our results provide a comprehensive example of how a *de novo*-originated gene organizes new regulatory circuits and thereby confers a selective advantage on *S. cerevisiae* to allow exquisite adaptation to the changing environment.

The origination of new genes provides pivotal genetic novelties for adaptive innovation of organisms^1^,^2^,^3^. Among all of the molecular mechanisms of gene origination, a number of protein-coding genes that have originated *de novo* from non-coding sequences have been shown to reshape the landscape of molecular evolution^4^,^5^,^6^. In particular, we previously reported that the *de novo* gene *MDF1* from baker's yeast plays a key role in regulating the yeast mating pathway by binding to MATα2^7^. *MDF1* was originally referred to as *FYV5*, performing a putative function in response to the K1 killer toxin^8^. Interestingly, we also observed that *MDF1* can promote growth, though the detailed mechanism of growth promotion was beyond the scope of our previous study^7^. The dual functions of *MDF1* in two pathways (mating and vegetative growth) indicate a pleiotropic role for this *de novo* gene. Sexual reproduction benefits a species by eliminating deleterious mutations through meiotic recombination, but mating is energetically costly for vegetative growth^9^. It has been shown that a growth-rate advantage can be gained by losing signaling at multiple points in the mating pathway^10^. However, there is little information available regarding the intrinsic molecular mechanism accounting for the trade-off of these two options. *MDF1* provides a unique opportunity for studying how two conflicting pathways are coordinated to maximize the adaptive advantage.

In the present work, we filled in the missing piece of the *MDF1* growth promotion puzzle and formed a comprehensive picture of how a *de novo* gene confers an evolutionary advantage by incorporating mating and growth pathways into one molecular network. We clearly demonstrated how *MDF1* enhances the effect of the active glucose signaling pathway to achieve advantageous vegetative growth in a rich glucose environment. The complete description of the dual functions of *MDF1* provides the first persuasive explanation for the dynamic crosstalk between mating and growth pathways from the aspect of a molecular mechanism.

## Results and Discussion

### Mdf1p shortens the lag phase of *S. cerevisiae*

**Figure 1:**
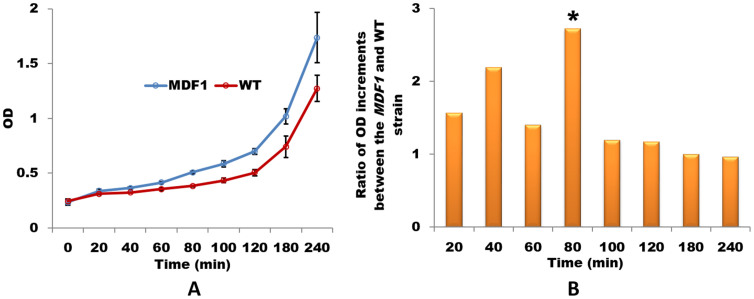
*MDF1* shortens the lag phase of *S. cerevisiae*. (A) Growth curves show that the *MDF1* strain driven by its own promoter exhibits a shortened lag phase in YPD medium compared with the wild-type strain. OD values were measured every 30 min for the first two hours. The mean and standard errors were represented in the growth curves. (B) There was a significant increase in the growth rate at 80 min in the *MDF1* strain compared with that in the wild-type strain (* indicates Two-tails T-test *P-value*<0.01).

Our previous results showed that Mdf1p is a newly originated key regulator of the mating pathway that acts by binding MATα2 and promoting vegetative growth in *S. cerevisiae*^7^. However, the molecular mechanism underlying how *MDF1* promotes growth was beyond the scope of our previous study. In this study, to investigate this question in depth, we therefore measured the growth rate of yeast cells every 20 min until growth plateau was reached. Then, we compared OD values between a wild-type strain and an *MDF1* strain in which expression was driven by the *MDF1* promoter (Methods). Interestingly, in the first two hours, the *MDF1* strain significantly outperformed the wild-type strain (Fig. 1A). Thereafter, the growth curves of the *MDF1* and wild-type strains paralleled with each other (Supplementary Fig. 1A), indicating that the growth acceleration induced by *MDF1* may be restricted to the time window of the first two hours. Indeed, *MDF1* began to be expressed at approximately 1 h and reached a peak at approximately 1.5 h, but the expression level of *MDF1* was reduced after 2 h (Supplementary Fig. 1B). The fluctuation of the expression of *MDF1* was synchronized with a significant increase in the OD at 80 min (Two-tails T-test, *P-value*<0.001; Fig 1B) in the growth curve, implying a causal link between *MDF1* and the growth rate. These results suggest that *MDF1* mainly exerts its function in the lag phase when yeasts are transferred to fresh fermentative medium.

### Mdf1p inhibits Snf1p to promote fermentation

**Figure 2:**
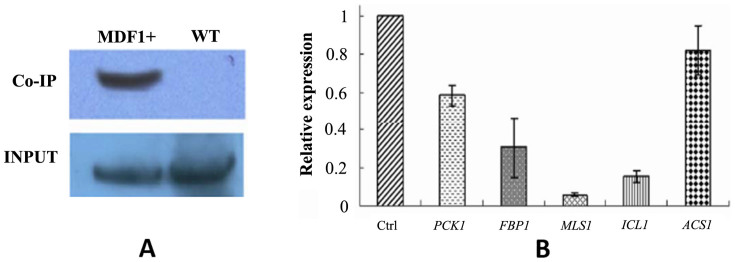
Inhibition of Snf1p by Mdf1p is responsible for the promotion of fermentation. (A) A Co-IP assay using an Mdf1p-3HA-tagged overexpression strain confirmed that Mdf1p and Snf1p interact in lag phase. The resultant Co-IP products were tested via western blotting using a Snf1p antibody, and 10% yeast samples before precipitation were used as an input control. (B) The expression levels of all of the glucose-repressed genes in the *MDF1* strain were reduced in comparison with the wild-type strain in quantitative RT-PCR assays. *ACT1* was used as an internal control.

When yeast cells are inoculated into fresh medium, they initially enter a brief lag phase during the first two hours, which is a crucial time for yeast cells to acclimatize to the new environment^11^. In the lag phase, the yeast cell experiences a delay or a pause of growth on the surface, but rapid adjustment of the yeast physiology to the fresh medium is in fact taking place to ensure optimal growth initiation^12^. The physiological and metabolic changes that occur during the lag phase are thought to prepare for the upcoming fermentation process because hundreds of fermentation-related genes are up-regulated in the genome^13^. Having established that *MDF1* shortens the lag phase, we hypothesized that *MDF1* may enhance fermentation. Indeed, there was no expression of *MDF1* detected under non-fermentative conditions in our experiments (Supplementary Fig. 2A), which was consistent with our previous observation that the *MDF1* strain presents no growth superiority in non-fermentable medium^7^. To confirm that *MDF1* functions in fermentative medium, our next task was to reveal the molecular mechanism underlying the enhancement of fermentation by *MDF1* during the lag phase. Co-IP assays were conducted using the *MDF1*-overexpressing strain to search for the factor(s) interacting with Mdf1p. Interestingly, a potential interaction partner, Snf1p, was repeatedly identified via MALDI-TOF/TOF (expect *P-value* <1.3 × 10^−10^) (Supplementary Figure 2B). To verify the interaction between Mdf1p and Snf1p, the products of the Mdf1p Co-IP assay were tested using a Snf1p antibody in vivo. Western blotting revealed the existence of Snf1p in the Co-IP products (Figure 2A), which firmly demonstrated that Mdf1p interacts with Snf1p in the lag phase. The interaction was further validated in a yeast two-hybrid assay, in which Mdf1p was fused to the DNA-binding domain of Gal4 (DB) and Snf1p to the activation domain of Gal4 (AD) (Supplementary Figure 2C). Taken together, the results obtained demonstrate the interaction between Mdf1p and Snf1p.

Snf1p is a governing factor in the transition from fermentative to respiratory growth in *S. cerevisiae*^14^,^15^. Snf1p leads to efficient de-repression of downstream glucose-repressed genes when glucose becomes limited^16^. Yeast cells thereby switch from fermentative growth to respiratory growth to utilize alternative carbon sources^17^,^18^. Given that Mdf1p facilitates fermentation, whereas Snf1p guides the non-fermentative pathway, we hypothesized that Mdf1p imposes negative regulation on Snf1p. To determine whether the Mdf1p-Snf1p interaction could inhibit the activity of Snf1p, we measured the expression levels of glucose-repressed genes, including *ICL1*, *FBP1*, *PCK1*, *ACS1*, and *MLS1*, which are activated by Snf1p^16^. All of the glucose-repressed genes displayed decreased expression levels in quantitative RT-PCR assays conducted in the *MDF1* strain (Fig. 2B), suggesting that the binding of Mdf1p and Snf1p produces an inhibitory effect on glucose-repressed genes. Taken these findings together, we proposed that the newly evolved *MDF1* gene integrated into the yeast glucose signaling pathway to speed up glucose utilization and fermentative growth via physically suppressing Snf1p. Fermentation as a preferred mode of resource consumption has particularly important significance in promoting the growth of yeasts. It has been shown that under aerobic conditions with an ample available carbon source such as glucose, baker's yeast predominantly conducts fermentation to generate energy for rapid cellular growth, though the yield of ATP is lower than during respiration^19^,^20^. The speed of resource intake matters much more than the efficiency of energy production when glucose is sufficient. Therefore, by accelerating glucose consumption, Mdf1p puts yeasts in an advantageous position in competing with other microbes present in the same environment.

### *MDF1* physically connects the growth pathway with the mating pathway

**Figure 3:**
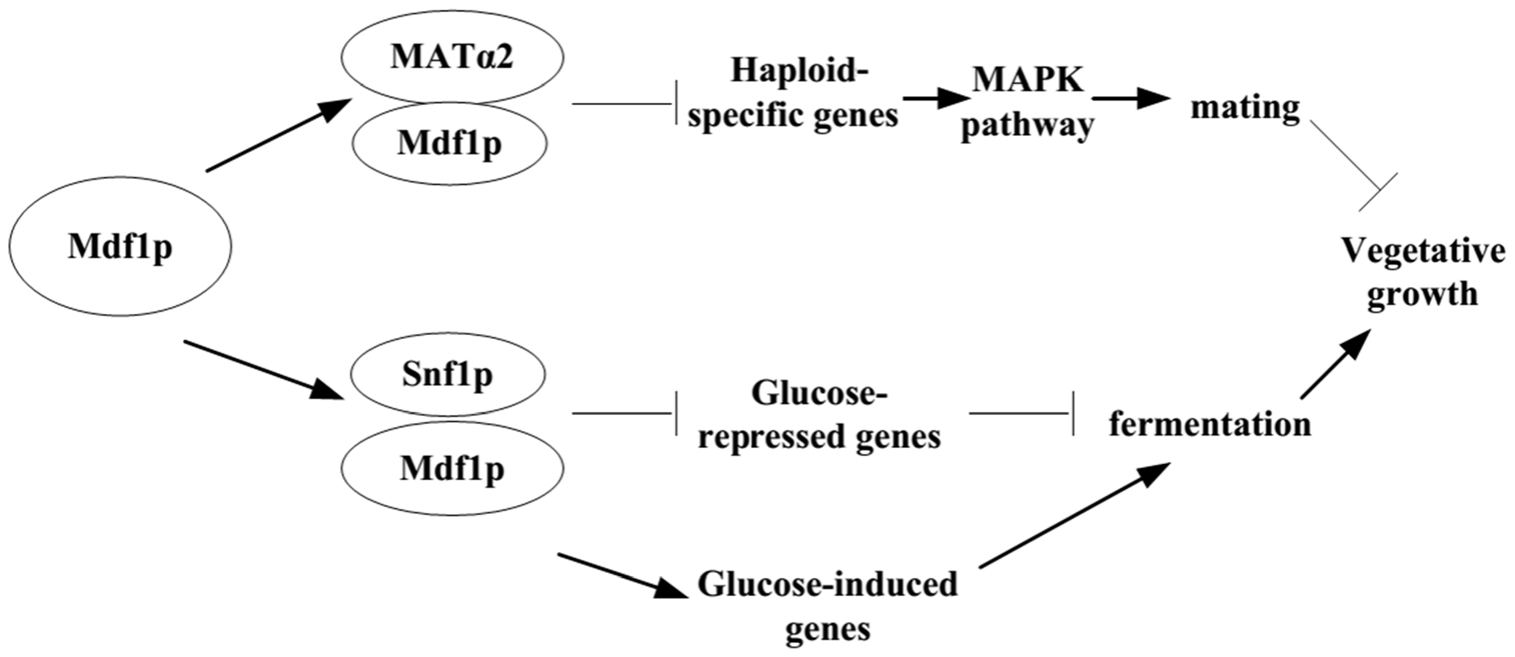
A model for the dual roles of *MDF1* in the mating and growth pathways. Promotion of vegetative growth can be achieved through two converging pathways: Mdf1p binds to the MATα2 protein to cooperatively inhibit haploid-specific genes that encode key components of the mating pathway (MAPK pathway) and thereby suppresses the mating behavior of budding yeast to limit the cost of mating, as revealed in a previous study by our group; and Mdf1p binds to Snf1p to turn off glucose-repressed genes and turn on glucose-induced genes, thus enhancing the activity of the glucose signaling pathway.

In our previous study, we revealed that Mdf1p was capable of suppressing yeast mating behavior by binding one of the yeast mating type determinants, MATα2, which would subsequently shut down the mating pathway^7^. In the present study, we discovered that Mdf1p could speed up vegetative growth through physically inhibiting the fermentation-suppressing factor Snf1p. Based on the evidence obtained thus far, we extended the previously proposed model^7^ concerning the established functions of Mdf1p in the mating and growth pathways (Figure 3): initially, especially after the recovery of haploid cells from growth arrest under unfavorable conditions, vegetative proliferation is advantageous for rapid resource consumption. Mdf1p enhances the effect of activating the glucose signaling pathway to speed up glucose metabolism and simultaneously suppresses the activity of the opposing mating pathway to limit the cost of mating.

Yeasts can reproduce both sexually and asexually. Natural selection can favor either vegetative fitness or the mating ability under different conditions and a negative correlation between these two traits has been proposed^21^. The dual roles of Mdf1p in mating and growth may help to explain the molecular link between these two conflicting options. Additionally, by mediating the trade-off between mating and vegetative growth, *MDF1* potentially provides an example of antagonistic pleiotropy, in which a gene is beneficial in one condition, but detrimental in another^22^,^23^,^24^,^25^. A genome-wide survey of antagonistic pleiotropy genes was recently performed in yeasts^26^. The prevalence of antagonistic pleiotropy genes in the yeast genome was beyond previous expectations. Regulatory resolution at the transcriptional level was proposed to explain the antagonistic pleiotropy genes found in the genomes. The identification of protein-protein interactions is another key index of pleiotropic characteristics^27^,^28^. We found that Mdf1p could retain antagonistic pleiotropy by interacting with different partners in two functionally conflicting pathways, where it interacts with MATα2 to repress the mating pathway and binds to Snf1p to promote the growth pathway. Delineation of the functional evolution of the *MDF1* gene not only provides a vivid example of how a *de novo* gene can play pivotal roles in important biological processes, resulting in adaptation, but also demonstrates a novel molecular mechanism to explain the antagonistic pleiotropy between the mating and growth pathways according to a molecular mechanism.

## Methods

### Yeast strains and growth conditions

The strain of *S. cerevisiae* used for all genetic manipulations in this study was BY4742 (MATα his3Δ1 leu2Δ0 lys2Δ0 ura3Δ0), provided by Prof. Jin-Qiu Zhou of the Shanghai Institutes for Biological Sciences. Cells were grown in standard fermentative YPD medium containing 1% yeast extract, 2% peptone, and 2% glucose at 3°C. For the marker-bearing strains, SD-Ura, SD-Trp or SD-His^29^ was used for selection.

### Growth rate assay

The details of the generation of the *MDF1* strain driven by its own promoter (previously referred to as the M+A-strain) were described by Li *et al*.^7^. Growth curves for the yeast strains were generated using a Bio photometer (Eppendorf). The cultured *MDF1* strain and the wide-type strain were diluted to 0.1 ± 0.01 OD independently. OD values were measured every 30 min for the first two hours and then every 1 h until 4 h. The experiments were repeated five times. Student's t-test was used to test the difference of growth rates among multiple time-points.

### Co-immunoprecipitation assays

A total of 100 OD yeast cells overexpressing either 6xHis or HA-tagged Mdf1p were lysed in 500 μl of lysis buffer (50 mM Tris-HCl (pH 7.5), 150 mM NaCl, 1 mM EDTA, 1 mM PMSF, protease inhibitor cocktail (Roche), 0.2 mM Na3VO4, 100 mM NaF, 0.2% NP-40) via beating with glass beads, followed by centrifugation at 12000 g for 5 min at 4°C. The 1 ml supernatant was pre-cleared with 5 μl of protein-G agarose (Santa Cruz). An anti-His or HA mouse monoclonal antibody (R&D systems) was added at a dilution of 1:100, followed by overnight incubation at 4°C with mild shaking. Then, 20 μl of protein-G agarose was added, followed by incubation for 1 h and centrifugation at 850 g for 10 min at 4°C. The beads were washed three times with lysis buffer and an additional three times with wash buffer. After the final wash, the beads were boiled for 5 min in 50 μl of SDS-PAGE loading buffer. As a negative control, wild-type yeast cells (100 OD) were also treated according to the method described above. A 20 μl immunoprecipitation sample obtained from the 6xHis-tagged Mdf1p strain and a control sample were analyzed via SDS-PAGE. The gel was visualized by silver staining with the PlusOneTM silver stain kit (GE healthcare). Compared with the control, the significant and repeatable unique band was analyzed through MALDI-TOF/TOF. The samples obtained from the HA-tagged Mdf1p strain and the control strain were analyzed by western blotting using an Snf1p antibody (Santa Cruz).

### Yeast two-hybrid assays

All procedures essentially followed the Yeast Protocols handbook (Clontech). Briefly, the coding sequence of *MDF1* was fused to the DNA-binding domain of Gal4 (DB), and the coding sequence of *SNF1* was fused to the activation domain of Gal4 (AD). Then, these two plasmids were co-transformed to identify interactions or were transformed separately as negative controls into the host strain Y190 (MATa, gal4-542, gal80-538, his3, trp1-901, ade2-101, ura3-52, leu2-3, 112, *URA3*::GAL1-LacZ, *LYS2*::GAL1-HIS3cyhr). After selection on SD-Trp-Leu-His plates, 5-Bromo-4-chloro-3-indoly-β-D-galactoside (X-gal) was added to evaluate the strength of the interaction.

### Quantitative RT-PCR

Quantitative RT-PCR primers were designed for the glucose-repressed genes, *ICL1*, *FBP1*, *PCK1*, *ACS1*, and *MLS1*, respectively. Quantitative RT-PCR assays were performed in triplicate in a 96-well plate in a Bio-Rad iCycler iQ Real-Time PCR Detection System (Bio-Rad) using iQSYBR Green Supermix (Bio-Rad), according to the manufacturer's recommended protocol. The CT values of each sample were acquired with iCycler iQ software 3.0 (Bio-Rad). Specific primers for *ACT1* were used as an internal control. The initial 150 bp of the coding sequence were employed to quantify each of the selected genes.

## Author Contributions

D.L., H.J. and W.W. designed the experiments. D.L., Y.Z. and L.L. performed most of the experiments and data analysis. D.L., Y.Z., H.J. and W.W. wrote the paper.

## Supplementary Material

Supplementary Information Supplement information
