# Supplementary material for: Pleiotropy of the de novo-originated gene MDF1
Source: Sci Rep. 2014 Dec 2;4:7280. doi: 10.1038/srep07280 (PMC4250933; doi:10.1038/srep07280)
Supplement: Supplementary Information — Supplement information [file srep07280-s1.docx]

**Pleiotropy of the *de novo*-originated gene *MDF1***

Dan Li^1,#^, Zhihui Yan^2^, Lina Lu^2^, Huifeng Jiang^2,*^, Wen Wang^1,*^

^1^State Key Laboratory of Genetic Resources and Evolution, Kunming Institute of Zoology, Chinese Academy of Sciences (CAS), Kunming, Yunnan 650223, People's Republic of China

^2^Key Laboratory of Systems Microbial Biotechnology, Tianjin Institute of Industrial Biotechnology, Chinese Academy of Sciences(CAS), Tianjin 300308, People's Republic of China

^#^Current address: Neural Dynamics Laboratory, University of British Columbia 828 W. 10th Ave, Vancouver B.C., Canada V5Z 1L8

Supplementary Figures 1–2


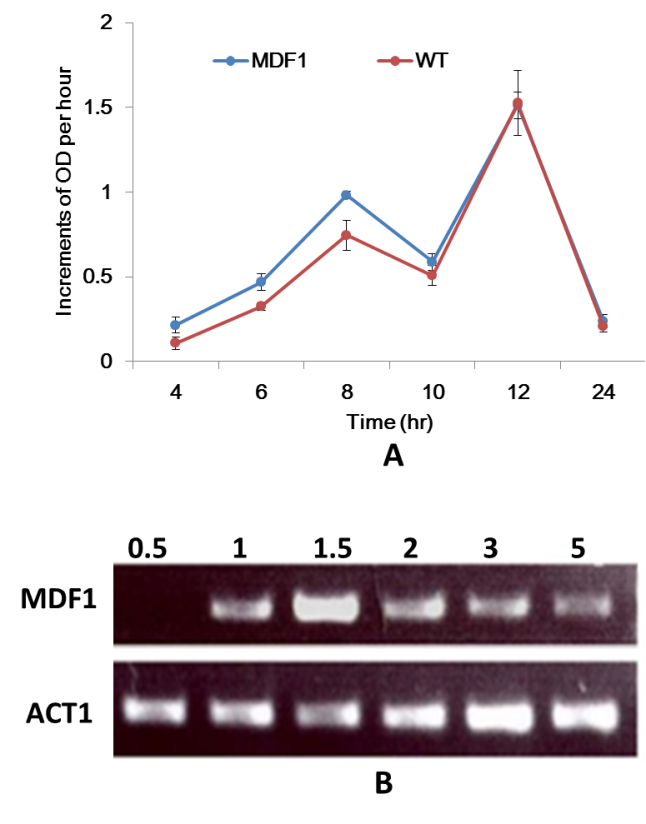


Supplementary Figure 1. (A) The growth curve of *MDF1* strain and WT strain within 24 h. There is no significant difference for growth rate between them. (B) The expression profile of *MDF1* gene at the first five hours. *ACT1* is an internal control.

**
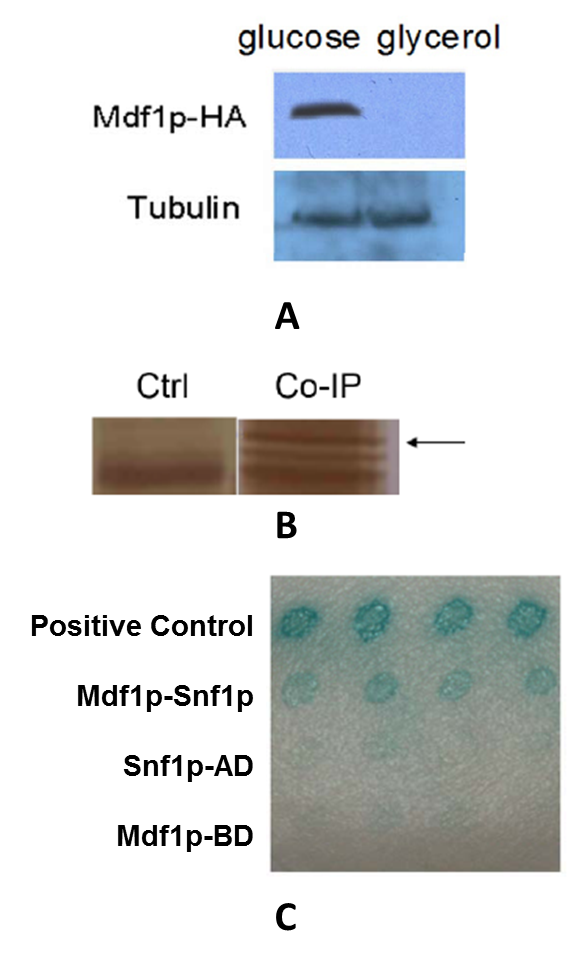
**

Supplementary Figure 2. (A) *MDF1* is translated into protein under fermentative condition (glucose), but no protein can be detected under non-fermentative condition (glycerol). Endogenous Mdf1p tagged with 3HA was detected by western blotting, and tubulin was used as a positive control. (B) A potential Mdf1p’s interaction partner Snf1p was identified by Co-IP assay. Wild type strain (Ctrl) was used as negative control, and Mdf1p overexpression strain was used for Co-IP assay. Two independent experiments were performed, and the repeatable band of Snf1p is pointed with arrow. (C) The yeast two-hybrid assays show that Mdf1p interacts with Snf1p in vivo. P53-SV40 as the positive control; Snf1p fused with the activation domain of *GAL4* (AD) and Mdf1p fused with the DNA-binding domain of *GAL4* (DB) as negative control. Four independent clones were patched in the selective plates.
